# PACS plus criteria: a retrospective cohort review of 612 consecutive patients treated with bilateral YAG peripheral iridotomies

**DOI:** 10.1038/s41433-023-02626-5

**Published:** 2023-06-20

**Authors:** Su Ling Young, Kelvin K. W. Cheng, Niamh O’ Connell, Roshini Sanders, Pankaj K. Agarwal

**Affiliations:** 1 Princess Alexandra Eye Pavilion, NHS Lothian, Edinburgh, UK; 2 Department of Ophthalmology, University of Edinburgh, Edinburgh, UK; 3 Queen Margaret Hospital, NHS Fife, Dunfermline, UK

**Keywords:** Outcomes research, Risk factors

## Abstract

**Introduction:**

With an ageing population and better life expectancy, the prevalence of angle closure disease is expected to increase by 20% per decade. In 2022, the Royal College of Ophthalmologists (RCOphth) issued a guideline on managing angle closure disease. Hospital eye service (HES) referral and prophylactic treatment are recommended only for primary angle closure suspect (PACS) with “Plus” features only. We aimed to examine patients previously treated with YAG peripheral iridotomies (YAG PI) for the presence of “PACS Plus” features.

**Methods:**

A retrospective cohort study of consecutive patients treated with YAG PI between 2015 and 2019 at a tertiary referral NHS eye centre was reviewed. Cases were examined to identify and classify patients into Primary Angle Closure (PAC), PACS, and Primary Angle Closure Glaucoma (PACG). Patients with PACS were studied for “Plus” features.

**Results:**

Six hundred twelve patients with gonioscopy-confirmed angle closure (defined as a minimum 180 degrees iridotrabecular contact) treated with YAG PI from years 2015 to 2019 were included in the analysis. The mean age of patients presenting with angle closure disease was 68.5 years (SD 11.3). There were 390 (63.7%) patients with PACS, 102 (16.6%) with PAC and 120 (19.7%) with PACG. Of the PACS patients, 159(40.8%) patients had no “Plus” features. 181 (40.2%) patients had 1 “Plus” feature, 37 (9.5%) had 2 “Plus” features and 13 (3.3%) patients had 3 “Plus” features.

**Conclusion:**

In our cohort, a considerable proportion (40.8%) of PACS patients treated with YAG PI did not have Plus features and therefore that would not meet the proposed criteria for HES referral and YAG PI. With the proposed guidance, we expect a considerable reduction in HES referrals. Nonetheless, community optometry services should be supported and trained to provide monitoring for patients with PACS not referred to the HES.

## Introduction

With an ageing population and better life expectancy, the prevalence of angle closure disease is expected to increase by 20% per decade [1]. It is recognised that angle closure, defined as 180 degrees or more of iridotrabecular contact (ITC), is a spectrum, where primary angle closure suspect (PACS) constitutes the earliest presentation of disease. Primary angle closure (PAC) is termed when raised IOP, or features suggestive of trabecular obstruction by the peripheral iris, such as peripheral anterior synechiae, elevated intraocular pressure, iris whirling (distortion of the radially orientated iris fibres), “glaucomflecken” lens opacities, or excessive pigment deposition on the trabecular surface is noted in addition to PACS [2]. Primary angle closure glaucoma (PACG) is termed when patients with PAC have glaucomatous optic atrophy and corresponding visual field loss [2].

A shallow anterior chamber is well-recognised as a risk factor for the development of angle closure disease. Increasing age [3], race [4] and female gender [5] are other recognised risk factors for PACG. In 2022, the Royal College of Ophthalmologists(RCOphth) published guidelines on managing angle closure disease [6] which recommended hospital eye service referral and prophylactic treatment only for PACS patients with “Plus” features. PACS is defined using the angle criteria of [1] a limbal chamber depth grade of <0.25 or [2] an anterior segment OCT showing irido-trabecular contact (ITC). “Plus” risk factors are defined as follows:

- People with only one “good eye” in which deterioration of vision may threaten independent living or livelihood
- Vulnerable adults who may not report ocular or vision symptoms
- Family history of significant angle closure disease
- High hypermetropia (>+ 6.00 dioptres)
- Diabetes or another condition necessitating regular pupil dilation
- Use of antidepressants or medication with an anticholinergic action
- People either living in remote locations (such as foreign aid workers, armed forces stationed overseas or oil rig workers etc.) where rapid access to emergency ophthalmic care is not possible.

The Zhongshan Angle Closure Prevention (ZAP) study, a large single-centre randomised controlled trial of 889 PACS individuals treated with prophylactic laser iridotomy (PI) [7]. Participants were randomised to observation versus YAG PI and were followed up for 72 months to evaluate incident primary angle closure disease as a composite endpoint of elevation of intraocular pressure, peripheral anterior synechiae, or acute angle closure as a significant primary outcome during 72 months of follow-up. The study demonstrated a substantial decrease in the incidence of angle closure in 4.19 per 1000 eye-years in treated eyes compared with 7.97 per 1000 eye-years in untreated eyes (hazard ratio 0.53; 95% CI 0.30–0.92; *p* = 0.024). Although statistically significant, this was not deemed clinically significant. With cautious extrapolation to prevention of glaucoma, the number needed to treat (NNT) to prevent a case of PACG is 126.

It is also important to note the higher risk of PAC in Chinese eyes [8, 9], extrapolating this to the UK population would likely increase the NNT by 2–3 fold based on the ratio of acute angle closure occurring in Caucasians compared to East Asians [10]. In addition, laser PI has been associated with greater pre-procedure anxiety and pain by patients [10].Therefore, prophylactic laser peripheral iridotomy for primary angle-closure suspects without “Plus” features is no longer recommended in the new guidance by the RCOphth.

Social deprivation has been associated with a greater severity of glaucoma at presentation [11] and previous work from this group had demonstrated patients from lower socioeconomic backgrounds were more likely to present with acute angle closure [12]. Late presentation is often reported to the primary reason behind the greater severity of glaucoma at presentation. Associations between socioeconomic deprivation and a higher prevalence of type 2 Diabetes [13], shorter axial length [14] and learning disability [15] have also been described.

In this study, we aimed to examine consecutive angle closure patients treated with bilateral YAG peripheral iridotomies at a tertiary ophthalmology unit in the UK for the presence of PACS Plus features. We also aimed to examine the association between PACS Plus Risk Factors and socioeconomic deprivation.

## Methods

This is a retrospective review of 612 consecutive patients with angle closure disease treated with bilateral YAG PI at a tertiary university hospital. This tertiary unit possessed two laser machines capable for YAG PI, and all procedures performed on the machines are documented in a paper hospital laser logbook. This hospital laser logbook was reviewed by two authors to identify all consecutive patients that underwent YAG PIs between 01/01/2015 and 31/12/2019. Electronic and paper case notes for corresponding patients were then identified and examined to determine the severity of angle closure disease and for the presence of PACS Plus criteria in patients with PAC, PACS and PACG. Age, sex, better/only eye status, refractive error, use of antidepressants, family history of glaucoma, presence of diabetes, vulnerable adult status and address were identified from electronic medical record systems. Ocular clinical data such as the clinical diagnosis of PAC, PACG,or PACS and visual field mean deviation and pattern deviation at presentation were identified from review of paper-based clinical records. In this study we defined a positive family history as a 1st degree or 2nd relative with glaucoma. We defined vulnerable adult status by learning or cognitive disability meeting criteria for incapacity or requiring full time care (eg: nursing home care). Patients were considered to live in a remote location if clinical documentation describes limited access to healthcare due to overseas work or if the registered address was location beyond 3 h travel of a dedicated ophthalmology unit. Patient consent for all included patients was obtained at time of laser procedure for data collection and analysis. The study received ethics approval from the local quality improvement and audit committee.

The Scottish Index of Multiple Deprivation (SIMD) is a relative measure of deprivation of the Scottish population by postcode area. The SIMD highlights the extent of deprivation across seven domains: income, employment, education, health, access to services, crime and housing. The SIMD quintiles divide the Scottish population into 5 quintiles, with the first quintile “1” representing most deprived areas and quintile “5” least deprivated. Patients’ postcodes were matched with SIMD quintiles using the Scottish government’s SIMD database [16] to provide a relative measure of deprivation for statistical analysis.

Statistical analysis was performed using the SPSS 27 Package for Statistical Analysis (IBM, Massachusetts, USA). Differences between the PAC, PACG and PACS groups were compared using the *t*-test, Mann–Whitney U and a chi-squared test for normally distributed, non-normally distributed and categorical data, respectively. Univariate and multivariate regression was performed to identify associations between levels of deprivation and the PACS plus factors.

This study received ethics approval from the local quality improvement team.

## Results

Six hundred and twelve patients were included in this study. The majority of patients had PACS, followed by PACG and PAC. Their baseline clinical characteristics are summarised in Table 1.

**Table 1:** Characteristics of patients in the PAC, PACG and PACS groups.

	PACS (*n* = 390)	PAC (*n* = 102)	PACG (*n* = 120)
Age	67.3 (12.4)	68.3 (11.5)	72.7 (10.5)^*^
Sex (Female), *n* (%)	266 (68.2)	66 (64.7)	69 (57.5)^*^
Better/Only eye affected, *n* (%)	7 (1.8)	6 (5.9)^*^	7 (5.8)^**^
Vulnerable individuals, *n* (%)	8 (2.1)	2 (2.0)	12 (10.0)^***^
Positive family history, *n* (%)	110 (28.2)	30 (29.4)	17 (14.2)^**^
High hypermetropia, *n* (%)	52 (13.3)	20 (19.6)	32 (26.7)^**^
Diabetes, *n* (%)	41 (10.5)	18 (17.6)	15 (12.5)
Antidepressant use, *n* (%)	77 (19.7)	23 (22.5)	26 (21.7)
Remote location patients, *n* (%)	0	0	0
Patients with ≥ 2 PACS plus factors, *n* (%)	50 (12.9)	23 (22.5)^*^	28 (23.3)^**^
Number of PACS plus factors per patient			
0	159 (40.7)	37 (36.3)	45 (37.5)
1	181 (46.4)	42 (41.2)	47 (39.2)
2	37 (9.5)	15 (14.7)	22 (18.3)
3	13 (3.4)	5 (4.9)	6 (5.0)
4	0	3 (2.9)^*^	0

*Denotes *p* < 0.05, **denotes *p* < 0.01, ***denotes *p* < 0.001 compared to PACS group.

All data are presented as percentages apart from age, which is presented as mean (SD). Patients in the PAC and PACG groups were compared against the PACS group using a *t-*test, Mann–Whitney U and a chi-squared test for normally distributed, non-normally distributed and categorical data, respectively.

*p < 0.05; **p < 0.01; ***p < 0.001 versus patients in PACS group.

The mean spherical equivalent of the patients’ refractive error is presented in Supplementary Table 1. *Link to* Supplementary Table 1*:*

Comparing PACG and PACS patients, statistically significant differences between the PACG and PACS groups were noted for age, gender, family history, vulnerable status and high hyperopic status. Patients with PACG were older than patients with PACS. PACG patients had on average a mean deviation of −7.48 dB(7.5 dB) and pattern standard deviation of 4.54 dB (3.76 dB) in their worse eye on presentation. There were more females in all categories of angle closure disease. A greater proportion of PACG patients were vulnerable adults, hyperopic and had diseases affecting their better/only eye. A significantly higher proportion of PAC and PACG patients had two or more PACS plus risk factors compared to PACS patients. However, 35–38% of patients with PAC or PACG did not possess any PACS plus factors across all categories. A positive family history was noted more in PACS patients compared to PACG.

**Fig. 1:**
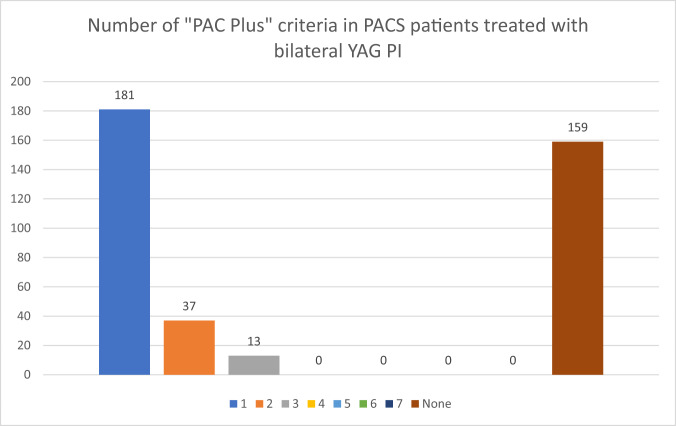
Number of PACS plus criteria in PACS patients treated with Bilateral YAG PI.

Examining PACS patients, there were more female compared to male patients. The majority of patients (46.4%) possessed 1 PACS plus risk factor, followed by 40.7% of patients who did not have any PACS plus risk factors. Examining the frequency of PACS plus factors in PACS patients, family history of glaucoma/angle closure, antidepressant usage and high hypermetropia (>+6.00D) were the commonest risk factors. The distribution of PACS plus criteria in PACS patients is demonstrated in Fig. 1.

On multivariate regression, antidepressant use was significantly associated with socioeconomic deprivation (Coefficient 1.09, *P* < 0.001). Other Plus features were not associated with socioeconomic deprivation and were not included in multivariate regression.

## Discussion

To our knowledge, this is the first study examining the presence of PACS plus criteria in angle closure patients treated with bilateral YAG PI. Our findings on the practice of YAG PI are consistent with other published studies. A previous UK survey in 2005 reported that 74.7% of consultant ophthalmologists would perform prophylactic YAG PI in eyes with PACS [17].

We observed that among PACS patients, more females than men in our cohort of patients were treated with bilateral YAG PI. This mirrors findings from other studies [5, 8], which supports a higher prevalence of angle closure in women.

Our data demonstrates that more PAC and PACG patients possessed two or more PACS Plus criteria compared to PACS patients. There were more vulnerable patients with PACG compared to PACS (*p* < 0.001). This may be due to delays in presentation to healthcare services due to the inability to report symptoms or barriers to accessing community healthcare. Subsequently, this leads to patients presenting at a later in the disease spectrum. Our findings support the utility of the PACS plus criteria for HES referral and prophylactic PIs in PACS patients with “plus” criteria. However, it should be noted that 30–40% of patients in all categories did not have any PACS plus risk factors. Therefore, even in the absence of risk factors, detailed assessment and monitoring for PAC and PACG are still important.

There is a fine line between conversion from PACS to PAC. As reported in the ZAP trial, the most common endpoint found in patients who converted into PAC from PACS was peripheral anterior synechiae (PAS) [7]. In Scotland, glaucoma suspect patients are entitled to free annual eye examinations under the General Ophthalmic Services (GOS) contract. Current tests such as Van Hericks and anterior segment OCT’s although are a reasonable test in predicting angle closure but not in identifying PAS. PAS can only be diagnosed with indentation gonioscopy and therefore community optometrists (at least one in each practice) should be trained in gonioscopy so that they are competent in detecting change from PACS to PAC.

The new guideline for angle closure does not address another major risk factor for acute angle closure-the age of the patient. Various studies have highlighted the role of enlarging lens and anterior depth reduction with age in the pathogenesis of angle closure in an elderly population group [18], with age being associated with a higher risk of conversion to PACG [19, 20]. In this study we observed similar findings, patients with PACG were older which may be explained by the more advanced disease state due to later presentation. Hence, community optometrists will need to be cautious in implementing the above ‘plus factors’ criteria for elderly patients in whom an expanding lens may make the patient more susceptible to acute or chronic angle closure glaucoma.

Positive family history, antidepressant use and high hypermetropia were the most commonly identified PACS plus criteria across all groups. Of these factors, antidepressant usage is the sole modifiable risk factor, as opposed to genetic or anatomic predisposition. We previously described inequalities in patients with angle closure disease, noting that patients from lower socioeconomic backgrounds were more likely to present with acute angle closure [12]. Our analysis identified a strong association between lower socioeconomic class and depression, in keeping with published literature [21]. Considering one of the plus factors is antidepressant use, community optometrists will need to be informed with a list of all the antidepressant medications as they come from a non-medical background.

40.7% of PACS patients in this study did not have any PACS plus criteria for referral. With the new guidance, we anticipate a significant decrease in HES referrals and prophylactic bilateral YAG PIs for PACS. Consequently, the demand on NHS community optometry for screening and monitoring of PACS patients not referred to the HES will increase significantly. Community uptake of screening is therefore essential. Patterns of health-seeking behaviour vary by socioeconomic status and literacy. Muir et al. found that glaucoma patients with lower literacy had poorer adherence to treatment [22]. The new PACS plus referral guidelines do not directly consider socioeconomic deprivation (SED). Saxby et al. [12] suggest that the relationship between higher levels of deprivation and presentation with acute primary angle closure (APAC) is due to barriers related to healthcare utilisation. As the new guideline depends on patient engagement with community optometry, it is unlikely that health inequality in access to eye care will be minimised directly.

With the Covid-19 pandemic, studies report ~8–38% [23, 24] of patients forgoing healthcare at the potential expense of adverse complications. Of these patients, patients with lower education levels and chronic conditions were most likely to forgo care. In the context of resuming services post-Covid- 19 and guidance, backlogs and hospital waiting list delays will lead to increasing numbers of patients seeking care at community NHS optometry practices. As such it is essential that community optometrists are trained in gonioscopy (at least one per practice) in order to maximise access to community screening for angle closure and to minimise unnecessary referrals to hospital eye services.

Future research should focus on identifying risk factors for progression from PACS to PAC and PACG and the cost implications of the new change in practice to the NHS and community services.

## Conclusions

The new guidelines will transform the management of PACS, reducing HES referrals for prophylactic YAG PI and consequently increasing the demand for community screening. It is imperative that community awareness, community optometry training in gonioscopy and accessibility to community screening is optimised to support the change in practice as a result of the new guidance.

## Summary

### What is known

- Prophylactic YAG PI is no longer recommended for primary angle closure suspect patients with no risk factors.

### What this paper adds

- To our knowledge, this is the first study to examine the potential implications of the new guidelines on clinical NHS practice.
- More patients with PAC and PACG had two or more PACS Plus factors compared to PACS patients, demonstrating the utility of the PACS plus criteria.
- PACS patients with multiple PACS Plus criteria should be monitored closely for progression to PAC and PACG. Community optometry training in gonioscopy is recommended for early recognition of PAC including features such as PAS which may precede IOP increase. Community practitioners should also be aware of other risk factors for acute angle closure including increasing age.

### Supplementary information

Supplemental Table 1

## Data Availability

Raw data that supports the findings of this study are available from the corresponding author, upon reasonable request.

## References

[1] Day AC, Baio G, Gazzard G, Bunce C, Azuara-Blanco A, Munoz B (2012). The prevalence of primary angle closure glaucoma in European derived populations: a systematic review. Br J Ophthalmol.

[2] Foster PJ, Buhrmann R, Quigley HA, Johnson GJ (2002). The definition and classification of glaucoma in prevalence surveys. Br J Ophthalmol.

[3] Foster PJ, Alsbirk PH, Baasanhu J, Munkhbayar D, Uranchimeg D, Johnson GJ (1997). Anterior chamber depth in Mongolians: variation with age, sex, and method of measurement. Am J Ophthalmol.

[4] Quigley HA, Broman AT (2006). The number of people with glaucoma worldwide in 2010 and 2020. Br J Ophthalmol.

[5] Vajaranant TS, Nayak S, Wilensky JT, Joslin CE (2010). Gender and glaucoma: what we know and what we need to know. Curr Opin Ophthalmol.

[6] Royal College of Ophthalmologists. The Management Of Angle-Closure Glaucoma. 2022. Available at: https://www.rcophth.ac.uk/wp-content/uploads/2021/10/The-Management-of-Angle-Closure-Glaucoma-Clinical-Guidelines-Executive-Summary.pdf. Accessed 19/06/2023.

[7] He M, Jiang Y, Huang S, Chang DS, Munoz B, Aung T (2019). Laser peripheral iridotomy for the prevention of angle closure: a single-centre, randomised controlled trial. Lancet.

[8] Cheng JW, Zong Y, Zeng YY, Wei RL (2014). The prevalence of primary angle closure glaucoma in adult Asians: a systematic review and meta-analysis. PLoS ONE.

[9] He M, Foster PJ, Johnson GJ, Khaw PT (2006). Angle-closure glaucoma in East Asian and European people. Different diseases?. Eye.

[10] Foster PJ, Ng WS, Nolan WP, Tanner L, Gazzard G, Day AC (2022). Prevention of angle-closure glaucoma: balancing risk and benefit. Eye.

[11] Ng WS, Agarwal PK, Sidiki S, McKay L, Townend J, Azuara-Blanco A (2010). The effect of socio-economic deprivation on severity of glaucoma at presentation. Br J Ophthalmol.

[12] Saxby E, Cheng K, O’Connell N, Sanders R, Agarwal PK (2022). Is there an association of socioeconomic deprivation with acute primary angle closure?. Eye (Lond).

[13] Connolly V, Unwin N, Sherriff P, Bilous R, Kelly W (2000). Diabetes prevalence and socioeconomic status: a population based study showing increased prevalence of type 2 diabetes mellitus in deprived areas. J Epidemiol Community Health.

[14] Goverdhan S, Fogarty AW, Osmond C, Lockwood A, Anderson L, Kirwan JF (2011). Shorter axial length and increased astigmatic refractive error are associated With socio-economic deprivation in an adult UK cohort. Ophthal Epidemiol.

[15] Shifrer D, Muller C, Callahan R (2011). Disproportionality and Learning Disabilities: Parsing Apart Race, Socioeconomic Status, and Language. J Learn Disab.

[16] Scottish Government. Scottish Index of Multiple Deprivation 2020.2020. Available at: https://www.gov.scot/collections/scottish-index-of-multiple-deprivation-2020/. Accessed 19/06/2023.

[17] Sheth HG, Goel R, Jain S (2005). UK national survey of prophylactic YAG iridotomy. Eye.

[18] Papaconstantinou D, Georgalas I, Kourtis N, Krassas A, Diagourtas A, Koutsandrea C (2009). Lens-induced glaucoma in the elderly. Clin Inter Aging.

[19] Yoo K, Apolo G, Zhou S, Burkemper B, Lung K, Song B, et al. Rates and Patterns of Diagnostic Conversion from Anatomical Narrow Angle to Primary Angle-Closure Glaucoma in the United States. Ophthalmol Glaucoma. 2022;6:169-76. 10.1016/j.ogla.2022.08.016 PMC9978040 36058536

[20] Markowitz SN, Morin JD (1984). Angle-closure glaucoma: relation between lens thickness, anterior chamber depth and age. Can J Ophthalmol.

[21] Li W, Ruan W, Peng Y, Lu Z, Wang D (2021). Associations of socioeconomic status and sleep disorder with depression among US adults. J Affect Disord.

[22] Muir KW, Santiago-Turla C, Stinnett SS, Herndon LW, Allingham RR, Challa P (2006). Health Literacy and Adherence to Glaucoma Therapy. Am J Ophthalmol.

[23] Baggio S, Vernaz N, Spechbach H, Salamun J, Jacquerioz F, Stringhini S (2021). Vulnerable patients forgo health care during the first wave of the Covid-19 pandemic. Prev Med.

[24] Menon LK, Richard V, de Mestral C, Baysson H, Wisniak A, Guessous I (2022). Forgoing healthcare during the COVID-19 pandemic in Geneva, Switzerland - A cross-sectional population-based study. Prev Med.

