# Supplementary material for: PACS plus criteria: a retrospective cohort review of 612 consecutive patients treated with bilateral YAG peripheral iridotomies
Source: Eye (Lond). 2023 Jun 20;37(18):3834–8. doi: 10.1038/s41433-023-02626-5 (PMC10698168; doi:10.1038/s41433-023-02626-5)
Supplement: Supplementary file 1 — Supplemental Table 1 [file 41433_2023_2626_MOESM1_ESM.docx]

***Supplementary Table 1: Mean Spherical Equivalent (SD) for Right and Left eyes for patients in the PAC, PACG and PACS groups.***

|  | Mean Right eye Spherical Equivalent (SD) | Mean Left Eye Spherical Equivalent (SD) |
| --- | --- | --- |
| PAC | +1.95 (2.56) | +1.99 (2.55) |
| PACG | +1.42 (2.29) | +1.71(2.27) |
| PACS | +1.88 (2.47) | +1.88(2.52) |
